# Potential harms of emergency department thoracotomy in patients with persistent cardiac arrest following trauma: a nationwide observational study

**DOI:** 10.1038/s41598-023-43318-0

**Published:** 2023-09-25

**Authors:** Ryo Yamamoto, Masaru Suzuki, Junichi Sasaki

**Affiliations:** 1 Trauma Service, Department of Emergency and Critical Care Medicine, Keio University School of Medicine, 35 Shinanomachi, Shinjuku, Tokyo, 160-8582 Japan; 2 Department of Emergency Medicine, Tokyo Dental College, Ichikawa General Hospital, Chiba, Japan

**Keywords:** Outcomes research, Trauma

## Abstract

Emergency department thoracotomy (EDT) was incorporated into traumatic out-of-hospital cardiac arrest (t-OHCA) resuscitation. Although current guidelines recommend EDT with survival predictors, futility following EDT has been demonstrated and the potential risks have not been thoroughly investigated. This study aimed to elucidate the benefits and harms of EDT for persistent cardiac arrest following injury until hospital arrival. This retrospective cohort study used a nationwide trauma registry (2019–2021) and included adult patients with t-OHCA both at the scene and on hospital arrival. Survival to discharge, hemostatic procedure frequency, and transfusion amount were compared between patients treated with and without EDT. Inverse probability weighting using a propensity score was conducted to adjust age, sex, comorbidities, mechanism of injury, prehospital resuscitative procedure, prehospital physician presence, presence of signs of life, degree of thoracic injury, transportation time, and institutional characteristics. Among 1289 patients, 374 underwent EDT. The longest transportation time for survivors was 8 and 23 min in patients with and without EDT, respectively. EDT was associated with lower survival to discharge (4/374 [1.1%] vs. 22/915 [2.4%]; adjusted odds ratio [OR], 0.43 [95% CI 0.22–0.84]; *p* = 0.011), although patients with EDT underwent more frequent hemostatic surgeries (46.0% vs. 5.0%; adjusted OR, 16.39 [95% CI 12.50–21.74]) and received a higher amount of transfusion. Subgroup analyses revealed no association between EDT and lower survival in patients with severe chest injuries (1.0% vs. 1.4%; adjusted OR, 0.72 [95% CI 0.28–1.84]). EDT was associated with lower survival till discharge in trauma patients with persistent cardiac arrests after adjusting for various patient backgrounds, including known indications for EDT. The idea that EDT is the last resort for t-OHCA should be reconsidered and EDT indications need to be deliberately determined.

*Trial registration* This study is retrospectively registered at University Hospital Medical Information Network (UMIN ID: UMIN000050840).

## Introduction

Traumatic out-of-hospital cardiac arrest (t-OHCA) introduces dismal consequences with extremely unfavorable neurological outcomes^1–3^. A considerable number of patients with t-OHCA present in extremis with massive bleeding^1, 4^, thus emergency department thoracotomy (EDT) with aortic occlusion via cross-clamping has been incorporated into t-OHCA resuscitation as a temporal hemostatic procedure^5–7^. However, futility following EDT in patients with t-OHCA was demonstrated in various studies^7, 8^, therefore, EDT is usually performed to offer hope for the survival of moribund trauma victims, rather than to provide a validated treatment.

Current clinical practice guidelines for trauma patients suggest that EDT is considered when t-OHCA survival predictors exist, including penetrating injury, chest injury (particularly cardiac injury), presence of signs of life, and shorter cardiopulmonary resuscitation duration^5, 9^. These predictors were associated with favorable outcomes among patients who underwent EDT. However, no well-designed studies validated the obvious benefits of EDT than without EDT even in selected patients with such predictors^9–11^. Moreover, additional hemorrhage due to EDT procedures can reduce the chance of survival, and such potential harms have not been sufficiently examined. Considering the report on the potential superiority of less-invasive temporal hemostasis for t-OHCA by resuscitative endovascular balloon occlusion of the aorta (REBOA)^8, 12^, the idea of EDT as the last resort for t-OHCA probably needs to be revisited.

Accordingly, we examined the clinical outcomes of trauma patients with OHCA using a nationwide trauma registry to elucidate the balance between the benefits and harms of EDT for patients with t-OHCA. Patients suffering from persistent cardiac arrest following injury until hospital arrival were targeted because they would minimally benefit from EDT and manifest procedure-related risks^13^. We hypothesized that EDT would be associated with lower survival to discharge of trauma patients in whom cardiac arrest was diagnosed both at the scene and on hospital arrival.

## Methods

### Study design and setting

A retrospective cohort study was conducted using data from the Japan Trauma Data Bank (JTDB). The JTDB was established as a Japanese nationwide trauma registry in 2003 and is maintained by the Japanese Association for the Surgery of Trauma and the Japanese Association for Acute Medicine. The JTDB is participated by > 250 tertiary care centers across Japan, and data are entered into an online data collection portal by treating physicians or volunteer registrars designated by each hospital^14^. All collaborating hospitals obtained individual local institutional review board approval for conducting research with human subjects in accordance with the Declaration of Helsinki before study initiation. This study was approved by the Institutional Review Board at Keio University School of Medicine, Tokyo, Japan on August 31st, 2020 (application number: 20090087), that waived informed consent because of the anonymous nature of the data. The current study was reported in line with the Strengthening the Reporting of ​Cohort Studies in Surgery criteria^15^.

In Japan, emergency medical service (EMS) providers perform cardiopulmonary resuscitation (CPR) according to the Japanese CPR guidelines that are based on the American Heart Association and International Liaison Committee on Resuscitation guidelines. Most EMS providers are certified to obtain intravenous access and place advanced airways for patients with t-OHCA, but these resuscitative procedures are not mandated. No EMS personnel are authorized to perform invasive trauma life support interventions, such as intraosseous access or needle/tube thoracostomy^14^. Physician-staffed ambulances are available in some regions and are usually dispatched from a tertiary care center. Physicians on ambulances can perform more invasive procedures, including thoracotomy, depending on their equipment and skills, although surgical hemostasis for major bleeding is not feasible^14^.

Current practice in Japan recommends EDT for patients with t-OHCA who arrive at an emergency department (ED) without a palpable pulse^16^. However, an EDT indication is generally decided according to the mechanism of injury, presence of signs of life, or transportation time that usually represents prehospital CPR time because futility following t-OHCA is highly expected regardless of EDT^5, 9^. Additionally, non-surgical resuscitation, including transfusion and cardiac life support for non-traumatic OHCA, is often provided by emergency physicians because trauma surgeons are not always present in the hospital. REBOA is performed with fluoroscopy and/or ultrasound guide at the ED depending on the capability of emergency physicians^8^.

### Study population

We retrospectively reviewed data from the JTDB from 2019 to 2021. Trauma patients (1) aged ≥ 18 years, (2) with no measurable blood pressure at the scene and (3) on hospital arrival, which is defined as 0 mmHg of systolic blood pressure on arrival, were included. Patients with missing data on survival at discharge were excluded.

### Data collection and definitions

Prehospital data was collected by EMS personnel and in-hospital by treating physicians. The information from the database included age, sex, Charlson comorbidity index, dependency in the activity of daily living (ADL), mechanism of injury, vital signs at the scene and on hospital arrival, prehospital resuscitative procedures, such as intubation, fluid administration, and transfusion, the presence of a physician at prehospital, transportation time, presence of signs of life on hospital arrival, Abbreviated Injury Scale (AIS) score, Injury Severity Score (ISS), Revised Trauma Score, Probability of survival calculated by Trauma and Injury Severity Score, emergency hemostatic procedures, including surgery, angiography, and REBOA, amount of transfusion within 24 h after hospital arrival, days of ventilator use, length of intensive care unit (ICU) and hospital stay, and survival status at ED and hospital discharge. Additionally, the frequency of EDT for patients with persistent cardiac arrest following injury was calculated at each institution. Details of EDT indication and the hemodynamic status during and after the EDT were not available in the database.

The presence of signs of life on hospital arrival was determined by a treating physician with any of the following: pupillary response, spontaneous ventilation, extremity movement, or cardiac electrical activity. Conversely, signs of life at the scene were considered present with any of the following: heart rate > 0 bpm, respiratory rate > 0 breaths/min, or Glasgow Coma Scale > 3 at the scene. Transportation time was defined as the duration between EMS arrival at the scene and hospital arrival, which is equal to prehospital CPR time in this study because cardiac arrest was diagnosed on EMS arrival at the scene.

### Outcome measures

The primary outcome was survival to discharge. Secondary outcomes included days of ventilator use, length of ICU and hospital stay, frequencies of emergency hemostatic procedures, including surgery, angiography, and REBOA, and the amount of transfusion, including red blood cells, fresh frozen plasma, and platelets.

### Statistical analysis

The primary outcome was compared between patients treated with and without EDT. Inverse probability weighting (IPW) with propensity scores was performed to adjust background characteristics between groups with and without EDT^17^. The propensity score for the average treatment effect in the study population was developed using a logistic regression model fitted with generalized estimating equations (GEE) to estimate the probability of conducting EDT and account for within-institution clustering^18, 19^. Before GEE model development, patterns of missing data were evaluated with Little’s Missing Completely At Random test, and missing non-outcome values were replaced with a set of substituted plausible values by creating 5 filled-in complete data sets using multiple imputations by the chained equation method^20^. Estimated associations in each of the imputed data sets were averaged together to give overall estimated associations.

Relevant covariates for the propensity score calculation were selected from known or possible predictors for performing EDT and predicting clinical outcomes in patients with t-OHCA based on previous studies^18, 21^. These covariates included age, sex, Charlson comorbidity index, ADL, mechanism of injury, prehospital procedures (intubation, fluid administration, and transfusion), presence of a physician at prehospital, transportation time, presence of signs of life at the scene and on hospital arrival, and injury severity (ISS and AIS in each region)^1, 9, 22–25^. The discrimination ability of the propensity score was analyzed using the c-statistic^18^. The IPW analyses were performed as adjusted analyses in which the primary outcome was compared using the chi-square test. Secondary outcomes were evaluated with odds ratios (ORs) or compared using the Mann–Whitney U test.

Primary results were validated using five sensitivity analyses. First, generalized estimating equation analysis with the logit link function was used to adjust patient backgrounds and differences in quality of care between participating hospitals to validate results that were not dependent on the propensity score calculation^19^. Second, a multivariate logistic regression analysis on the original data was conducted to confirm that the results were not overestimated by within-institution clustering and missing value imputation: the logistic model used the frequency of EDT at each institution as institutional characteristics, as well as the same covariates for the propensity score calculation. Third, IPW was conducted by excluding patients with < 0.05 or > 0.95 propensity scores to avoid extreme weight^17^. Fourth, IPW was conducted in the original data before missing value imputation. Finally, IPW with propensity score was repeated, in which the injury severity was not used for score calculation by the GEE model^26^, considering that the severity of injury would not be determined on hospital arrival in most of the patients with t-OHCA.

Additionally, crude numbers of patients in each minute of transportation time were drawn with the survival status at discharge to elucidate the threshold of transportation time (prehospital CPR time) for conducting EDT on patients with t-OHCA. These numbers were shown after dividing patients into those with and without EDT.

Subgroup analyses were performed to investigate the relationships between EDT, clinical characteristics, and survival to discharge. Targeted subgroups were selected based on previous research regarding t-OHCA and IPW analyses of the primary outcome were repeated in each subgroup. Patients were divided by age (< 65 vs. ≥ 65 years), presence of signs of life on hospital arrival, mechanism of injury, presence of severe chest injury (AIS in the chest < 3 vs. ≥ 3), requirements of hemostatic surgery, and the use of REBOA.

Descriptive statistics are presented as a median (interquartile range) or a number (percentage). Results were presented as a standardized difference (< 0.1 considered insignificant) and a 95% confidence interval (CI)^18^. The hypothesis was tested on the primary and secondary outcomes, with a two-sided α threshold of 0.05 considered significant. All statistical analyses were conducted using the IBM Statistical Package for the Social Sciences Version 28.0 (IBM Corp., Armonk, NY) and R Version 4.0.2 (R Foundation for Statistical Computing, Vienna, Austria).

### Ethics approval and consent to participate

This study was approved by the Institutional Review Board at Keio University School of Medicine, Tokyo, Japan on August 31st, 2020 (Application No.: 20090087). Informed consent was waived because of the anonymous nature of the data.

## Results

### Patient characteristics

**Figure 1:**
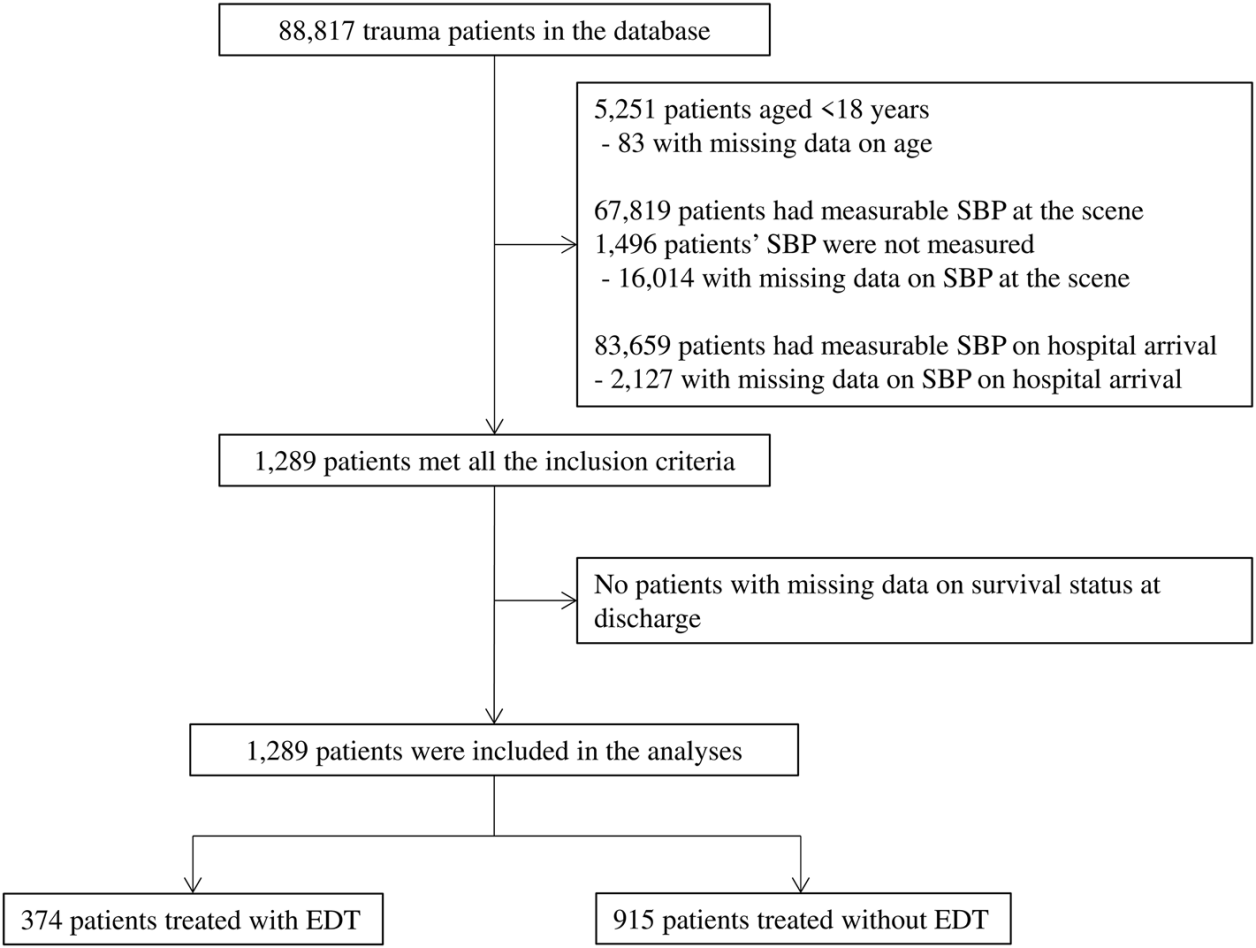
Patient flow diagram. Of the 88,817 trauma patients in the database, 1289 adults were transported with persistent cardiac arrest following injury until hospital arrival in 2019–2021, and thus eligible for this study. In total, 374 (40.9%) patients were treated with EDT (Fig. 1). Number of patients who did not meet each inclusion criterion was counted independently.

Of the 88,817 trauma patients in the database, 1289 adults were transported with persistent cardiac arrest following injury until hospital arrival in 2019–2021, and thus eligible for this study. In total, 374 (40.9%) patients were treated with EDT (Fig. 1).

Patient characteristics are shown in Table 1. The median transportation time (prehospital CPR time) was 10 min in both patients with and without EDT. Patients treated with EDT were younger; had higher ISS and AIS in the chest and the extremity/pelvis; and had lower AIS in the head compared to those treated without EDT. Additionally, a greater number of patients with EDT suffered from a blunt injury, were seen by a physician at prehospital, and had signs of life on hospital arrival. Conversely, the frequency of receiving prehospital therapeutic procedures and having signs of life at the scene was comparable between patients with and without EDT.

**Table 1:** Characteristics of patients with traumatic out-of-hospital cardiac arrest.

	Before IPW			After IPW		
	EDT	No EDT	Standardized difference	EDT	No EDT	Standardized difference
Case	374	915				
Age, years, median (IQR)	52 (35–69)	61 (42–77)	0.345	59 (41–74)	58 (40–76)	0.005
Sex, male, n (%)	268 (71.7%)	554 (60.9%)	0.228	790 (63.8%)	822 (64.1%)	0.001
Mechanism of injury, blunt, n (%)	341 (91.2%)	716 (78.3%)	0.365	1029 (83.0%)	1059 (82.0%)	0.025
Comorbidity, Charlson index, median (IQR)	0 (0–0)	0 (0–0)	0.275	0 (0–0)	0 (0–1)	0.000
Activity of daily living, independent, n (%)	207 (59.1%)	497 (59.3%)	0.003	665 (57.3%)	715 (60.3%)	0.035
Prehospital procedure, n (%)						
Intubation	45 (12.0%)	126 (13.8%)	0.052	148 (11.9%)	171 (13.2%)	0.055
Fluid administration	94 (25.1%)	265 (29.0%)	0.085	308 (24.8%)	357 (27.7%)	0.064
Transfusion	1 (0.3%)	2 (0.2%)	0.010	2 (0.2%)	2 (0.2%)	0.002
Physician presence at prehospital, n (%)	63 (16.8%)	116 (12.7%)	0.118	186 (15.0%)	179 (13.9%)	0.032
Signs of life, n (%)						
At the scene	13 (3.5%)	25 (2.7%)	0.043	31 (2.5%)	38 (2.9%)	0.027
On hospital arrival	25 (7.2%)	40 (4.8%)	0.101	62 (5.4%)	65 (5.6%)	0.002
Transportation time*, min, median (IQR)	10 (7–15)	10 (7–16)	0.066	10 (7–14)	10 (6–15)	0.007
ISS, median (IQR)	29 (18–45)	25 (18–36)	0.261	27 (17–41)	25 (20–41)	0.035
AIS, median (IQR)						
Head/neck	0 (0–3)	3 (0–5)	0.438	2 (2–4)	2 (2–4)	0.046
Face	0 (0–0)	0 (0–0)	0.000	0 (0–1)	0 (0–0)	0.046
Chest	4 (3–5)	3 (0–4)	0.649	3 (0–4)	3 (0–5)	0.098
Abdomen	0 (0–0)	0 (0–0)	0.283	0 (0–0)	0 (0–0)	0.029
Extremity/pelvis	2 (0–3)	0 (0–3)	0.228	2 (0–3)	0 (0–3)	0.099
Body surface	0 (0–0)	0 (0–0)	0.116	0 (0–0)	0 (0–0)	0.062
Revised Trauma Score, median (IQR)	0.00 (0.00–0.00)	0.00 (0.00–0.00)	0.018	0.00 (0.00–0.00)	0.00 (0.00–0.00)	0.065
TRISS Probability of survival, median (IQR)	0.02 (0.00–0.05)	0.02 (0.01–0.07)	0.029	0.01 (0.01–0.04)	0.02 (0.01–0.07)	0.074

EDT, Emergency department thoracotomy; IPW, Inverse probability weighting; IQR, Interquartile range; ISS, Injury severity score; AIS, Abbreviated injury scale; RTS, Revised trauma score; and TRISS, trauma and injury severity score. *Transportation time is equal to cardiopulmonary resuscitation time in this study.

After missing values were imputed (missing was not completely at random and suspected to be at random; Additional file 1), a propensity model of prediction to perform EDT was developed, and discrimination ability was calculated: c-statistic = 0.750 (0.722–0.778). Distribution of propensity scores before and after weighting was shown in Additional file 2. Table 1 shows the patient characteristics after IPW with standardized differences, wherein differences in covariates, including prehospital resuscitation and injury severity, were successfully attenuated using the propensity score (standardized difference < 0.1).

### Survival to discharge and secondary outcomes

**Figure 2:**
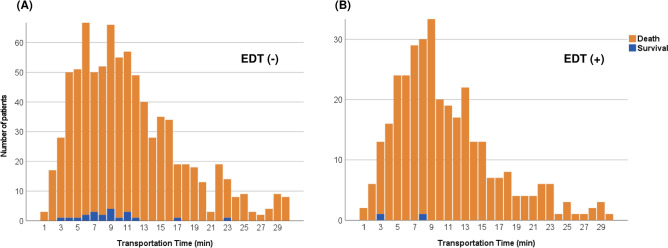
Number of patients with traumatic out-of-hospital cardiac arrest divided by transportation time. (**A**) Number of patients with traumatic out-of-hospital cardiac arrest (t-OHCA) treated without emergency department thoracotomy (EDT). (**B**) Number of patients with t-OHCA treated with EDT. Crude numbers of patients in each minute of transportation time were shown with survival status at discharge. The longest transportation time for survivors was 8 min in patients treated with EDT (**B**) and 23 min in those without EDT (**A**).

Survival to discharge was significantly lower among patients treated with EDT than among those without EDT (1.0% vs. 2.2%; OR: 0.43 [95% CI 0.22–0.84]; *p* = 0.011; Table 2). Sensitivity analyses with generalized estimating equations and multivariate logistic regression revealed similar findings; EDT was associated with reduced survival to discharge in patients with t-OHCA (OR: 0.08 [95% CI 0.01–0.71] and 0.06 [95% CI 0.01–0.76], respectively; Additional file 3). Additionally, IPW analyses with propensity score restriction and without the injury severity for propensity score calculation, as well as IPW using the original data without missing values imputation, similarly revealed a relationship between EDT and decreased survival to discharge (OR: 0.44 [95% CI 0.22–0.87], 0.41 [95% CI 0.21–0.78], and OR: 0.13 [95% CI 0.05–0.38], respectively; Additional file 3). Furthermore, crude numbers of patients in each minute of transportation time were shown in Fig. 2 and Additional file 4, which revealed 8 and 23 min as the longest transportation time for survivors in patients with and without EDT, respectively.

**Table 2:** Emergency department thoracotomy and clinical outcomes.

	EDT	No EDT	*p* value	OR (95% CI)
Survival to discharge				
Unadjusted, n/total (%)	4/374 (1.1%)	22/915 (2.4%)		
IPW, %	1.0%	2.2%	0.011	0.43* (0.22–0.84)
Survival without impaired neurological function, unadjusted, n/total (%)	4/374 (1.1%)	18/915 (2.0%)		
Emergency hemostatic procedures, % (95% CI)				
Surgery	46.0% (43.3–48.8%)	5.0% (3.8–6.1%)	< 0.001	16.39 (12.50–21.74)
Angiography	1.7% (1.0–2.4%)	1.1% (0.5–1.7%)	0.204	1.57 (0.78–3.17)
REBOA	5.7% (4.4–7.0%)	1.2% (0.6–1.7%)	< 0.001	5.17 (2.94–9.07)
Transfusion within 24 h after hospital arrival, U, mean, median (IQR)				
Red blood cell	3, 0 (0–4)	1, 0 (0–0)	< 0.001	
Fresh frozen plasma	2, 0 (0–2)	1, 0 (0–0)	< 0.001	
Platelets	1, 0 (0–0)	0, 0 (0–0)	0.002	
Days of ventilator use, mean, median (IQR)	1, 1 (0–1)	2, 1 (0–1)	< 0.001	
Length of ICU stay, days, mean, median (IQR)	0, 0 (0–1)	1, 0 (0–1)	0.154	
Length of hospital stay, days, mean, median (IQR)	1, 0 (0–1)	2, 0 (0–1)	< 0.001	

EDT, emergency department thoracotomy; OR, odds ratio; CI, confidence interval; IQR, interquartile range; ICU, intensive care unit; and REBOA, resuscitative endovascular balloon occlusion of the aorta. *Survival to discharge was compared between the two groups, in which no EDT was a referenced group.

EDT was associated with the higher frequency of hemostatic surgery and REBOA (OR: 16.39 [95% CI 12.50–21.74] and 5.17 [95% CI 2.94–9.07], respectively; Table 2), but not with the requirement of angiography. Moreover, the transfusion amount was higher in patients with EDT than those without EDT, whereas the length of ventilator usage and hospital stay was shorter among patients with EDT than those without EDT (Table 2).

### Subgroup analysis

Subgroup analyses (Table 3) revealed a relationship between lower survival to discharge and EDT in several subgroups: older patients (≥ 65 years), those with signs of life on hospital arrival and those without, those with blunt injury, and those with non-severe chest injury (chest AIS < 3). Conversely, younger patients (< 65 years), those with penetrating injury, and those with severe chest injury (chest AIS ≥ 3) had comparable survival regardless of EDT treatment.

**Table 3:** Survival to discharge in subgroup analyses.

	EDT	No EDT	OR	95% CI
Age				
< 65 years	1.2% (0.4–2.1%)	2.0% (1.0–3.0%)	0.62	0.27–1.42
≥ 65 years	0.6% (0.0–1.2%)	2.6% (1.2–3.9%)	0.22	0.60–0.77
Signs of life on hospital arrival				
( +)	4.9% (0.0–10.3%)	16.9% (7.8–26.0%)	0.25	0.07–0.96
(−)	0.5% (0.1–0.9%)	1.6% (0.9–2.4%)	0.28	0.10–0.76
Mechanism of injury				
Blunt	0.8% (0.3–1.4%)	2.4% (1.4–3.3%)	0.35	0.16–0.76
Penetrating	1.9% (0.1–3.8%)	3.0% (0.8–5.2%)	0.63	0.18–2.19
Severity of chest injury				
AIS in the chest < 3	0.8% (0.0–1.8%)	3.7% (2.0–5.4%)	0.22	0.06–0.75
AIS in the chest ≥ 3	1.0% (0.3–1.7%)	1.4% (0.5–2.2%)	0.72	0.28–1.84
Requirement of hemostatic surgery				
( +)	1.2% (0.3–2.1%)	20.3% (10.5–30.2%)	0.05	0.02–0.13
(−)	0.7% (0.1–1.4%)	1.3% (0.7–1.9%)	0.57	0.21–1.56
REBOA				
( +)	9.7% (2.9–16.6%)	26.7% (4.3–49.0%)	0.30	0.07–1.18
(−)	0.4% (0.1–0.8%)	1.6% (1.2–2.7%)	0.22	0.08–0.56

EDT, emergency department thoracotomy; OR, odds ratio; CI, confidence interval; AIS, Abbreviated Injury Scale; and REBOA, resuscitative endovascular balloon occlusion of the aorta. IPW analyses were performed in each subgroup.

Furthermore, patients who underwent hemostatic surgery and those treated without REBOA had significantly reduced survival to discharge when EDT was performed, whereas EDT was not associated with lower survival to discharge in those who did not undergo surgery and those treated with REBOA.

## Discussion

This study revealed that EDT was associated with lower survival to discharge in patients with persistent cardiac arrest following injury until hospital arrival. This result was obtained after adjusting for background characteristics, including known EDT indications in the clinical guidelines, such as the mechanism of injury, degree of intrathoracic injury, presence of signs of life, and prehospital CPR time.

Several pathophysiological mechanisms are considered for the study results. First, EDT might have introduced additional hemorrhage from an incision or iatrogenic lung/vascular injury, which mitigates the benefits of temporal hemostasis by aortic occlusion^13, 27^. A past study using a nationwide trauma database reported that extreme rapidness and suboptimal environment for EDT procedures caused complications in 15–30% patients^27^. Additionally, literature comparing EDT and REBOA as a technique of aortic occlusion suggested that less-invasive occlusion by REBOA had more survival benefits than EDT^8, 13^, and the current study revealed harmful effects of EDT only in patients who were not treated with REBOA. Additional bleeding by EDT would have affected the successful resuscitation of patients with t-OHCA.

Second, EDT could delay more efficient or definitive hemostasis, particularly in non-thoracic injuries. Aortic occlusion through EDT is achieved only within a few minutes, but such a short period is critical for vulnerable patients with persistent cardiac arrest after injury. Of note, patients in this study who underwent hemostatic surgery had a significantly higher survival rate (20.3%) when EDT was not performed. A previous study reported that > 10% of patients who underwent laparotomy following t-OHCA had favorable neurological outcomes^28^; thus, prompt hemostasis without aortic cross-cramping by EDT can be ideal in selected injuries.

Third, EDT would not provide therapeutic effects in patients who suffered from non-hemorrhagic cardiac arrests, such as severe traumatic brain injury, spinal cord injury, and traumatic asphyxia^29, 30^. EDT should be emphasized to impede non-surgical cardiac life support in such populations^31^. Given that EDT was performed in patients even with prolonged prehospital CPR time, an exclusion criterion for EDT in several guidelines, considerable number of patients would have undergone EDT inappropriately and harms would be manifested.

Patients who were treated with EDT underwent hemostatic surgery and REBOA more frequently and received higher amounts of transfusion. The survival rate was eventually lower in patients with EDT; thus, these differences would reflect iatrogenic additional hemorrhage due to EDT and/or inappropriate continuation of resuscitation, rather than an effective use of resources continued after EDT. Notably, the length of hospital stay and ventilator use was shortened for a day in patients who underwent EDT.

Subgroup analyses revealed that EDT caused no obvious harm to patients with penetrating injury or severe chest injuries, which is probably because intrathoracic injuries could be treated through thoracotomy. Moreover, EDT would be harmful, particularly to the elderly in this study. The elderly are vulnerable to insufficient organ oxygenation due to hemorrhage^32^; therefore, adverse effects of EDT would have become apparent in these patients. In addition, while the number of patients who had signs of life on hospital arrival was only 65 (0.5%), EDT was associated with lower survival rate regardless of presence of sings of life on hospital arrival. However, these results should be interpreted with caution due to the small sample sizes in the subgroups, particularly for the results with wide 95% CI.

Importantly, this study does not deny the usefulness of EDT for t-OHCA. Other t-OHCA expected to have more favorable clinical outcomes^9, 13^, such as OHCA successfully resuscitated at prehospital, may benefit from EDT because the targeted population included those with persistent cardiac arrest. However, the assumption that patients with t-OHCA lose chances of survival by not performing EDT should be reconsidered based on the current results. The potential harms of EDT should be validated in further studies.

Study results must be interpreted within the context of the study design. We investigated data at the JTDB, which does not record EDT indications. Therefore, our results could have been different if the decision to conduct EDT depended on unrecorded strong prognostic factors and further comprehensive understanding of the results cannot be achieved. Another limitation is the unavailable details of the hemodynamic status during EDT and intrathoracic injuries through EDT. Although additional hemorrhage related to the procedure and delayed definitive hemostasis outside the chest would be reasons for unfavorable outcomes following EDT, pathophysiological benefits and drawbacks were not measured in this study and therefore could not be validated based on objective data. Moreover, we investigated the consequences of EDT only in patients with t-OHCA because harmful effects are considered to influence this population the most. Our results cannot be generalized to patients with impending cardiac arrest or those in whom cardiac arrest is diagnosed at the hospital, such as during surgery and after ICU admission. Effects of EDT on these population should be further examined in another study. Furthermore, as this study is conducted in a specific region, Japan, with a certain trauma care system, the applicability of the findings to different healthcare systems and settings might be limited. Finally, unrecorded prognostic factors (e.g., actual time of cardiac arrest) or unmeasured/high-missing information related to severity can potentially introduce biases and impede the accuracy of results. Residual biases after IPW would also exist because different population, such as patients with and without signs of life, were compared in this study. Well-designed prospective study focusing on the timing, indication, and physiological consequences of EDT should be conducted to validate the current results.

In summary, EDT was associated with lower survival to discharge among trauma patients in whom cardiac arrest was determined both at the scene and on hospital arrival. EDT was related to more frequent hemostatic surgery, REBOA, and transfusion, although the risks of EDT would eventually outweigh the benefits of survival. The idea that EDT is the last resort for t-OHCA should be reconsidered and the indications for EDT need to be deliberately determined.

### Supplementary Information

Supplementary Legends.

Supplementary Figure S1.

Supplementary Figure S2.

Supplementary Table S1.

Supplementary Table S2.

## Data Availability

The data of this study are available from the Japanese Association for Trauma Surgery and the Japanese Association for Acute Medicine, but restrictions apply to the availability of these data, which were used under license for the current study, and so are not publicly available. Data are however available from the authors upon reasonable request to the corresponding author (Ryo Yamamoto) and with permission of the Japanese Association for Trauma Surgery and the Japanese Association for Acute Medicine.

## Abbreviations

EDT: Emergency department thoracotomy
t-OHCA: Traumatic out-of-hospital cardiac arrest
UMIN: University Hospital Medical Information Network
REBOA: Resuscitative endovascular balloon occlusion of the aorta
JTDB: Japan Trauma Data Bank
EMS: Emergency medical service
CPR: Cardiopulmonary resuscitation
ED: Emergency department
ADL: Activity of daily living
AIS: Abbreviated injury scale
ISS: Injury severity score
ICU: Intensive care unit
IPW: Inverse probability weighting
GEE: Generalized estimating equations
OR: Odds ratio
CI: Confidence interval
