# Supplementary material for: Potential harms of emergency department thoracotomy in patients with persistent cardiac arrest following trauma: a nationwide observational study
Source: Sci Rep. 2023 Sep 25;13:16042. doi: 10.1038/s41598-023-43318-0 (PMC10520031; doi:10.1038/s41598-023-43318-0)
Supplement: Supplementary file 1 — Supplementary Legends. [file 41598_2023_43318_MOESM1_ESM.docx]

**Additional files**

**Additional file 1:**

**Table S1. Missing values**

**Additional file 2:**

**Figure S1. Distribution of propensity scores**

Distribution of propensity scores before (A) and after (B) inverse probability weighting was shown. EDT, emergency department thoracotomy.

**Additional file 3:**

**Table S2. Survival to discharge in sensitivity analyses**

**Additional file 4:**

**Figure S2. Number of patients with traumatic out-of-hospital cardiac arrest divided by transportation time (whole population)**

Crude numbers of patients treated with and without emergency department thoracotomy were shown with survival status at discharge.
