# Supplementary figures and images for: Potential harms of emergency department thoracotomy in patients with persistent cardiac arrest following trauma: a nationwide observational study
Source: Sci Rep. 2023 Sep 25;13:16042. doi: 10.1038/s41598-023-43318-0 (PMC10520031; doi:10.1038/s41598-023-43318-0)

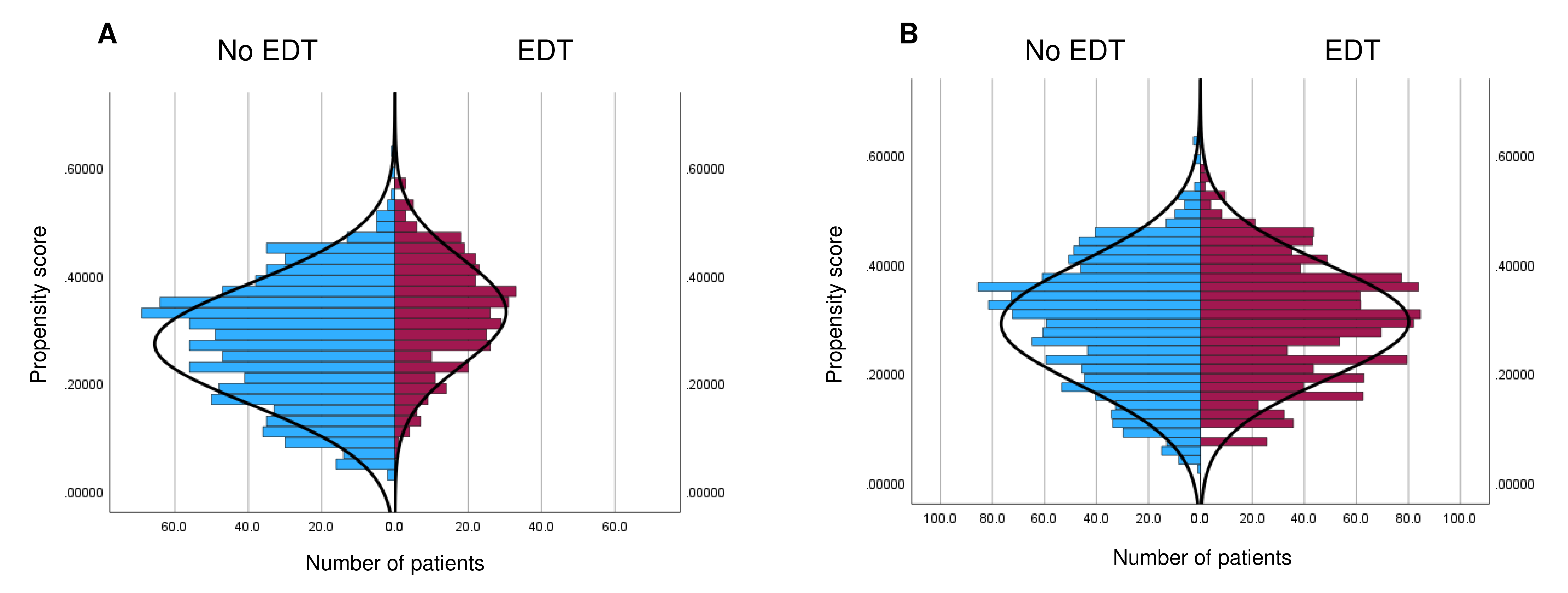

Supplement: Supplementary file 2 — Supplementary Figure S1. [file 41598_2023_43318_MOESM2_ESM.tiff]

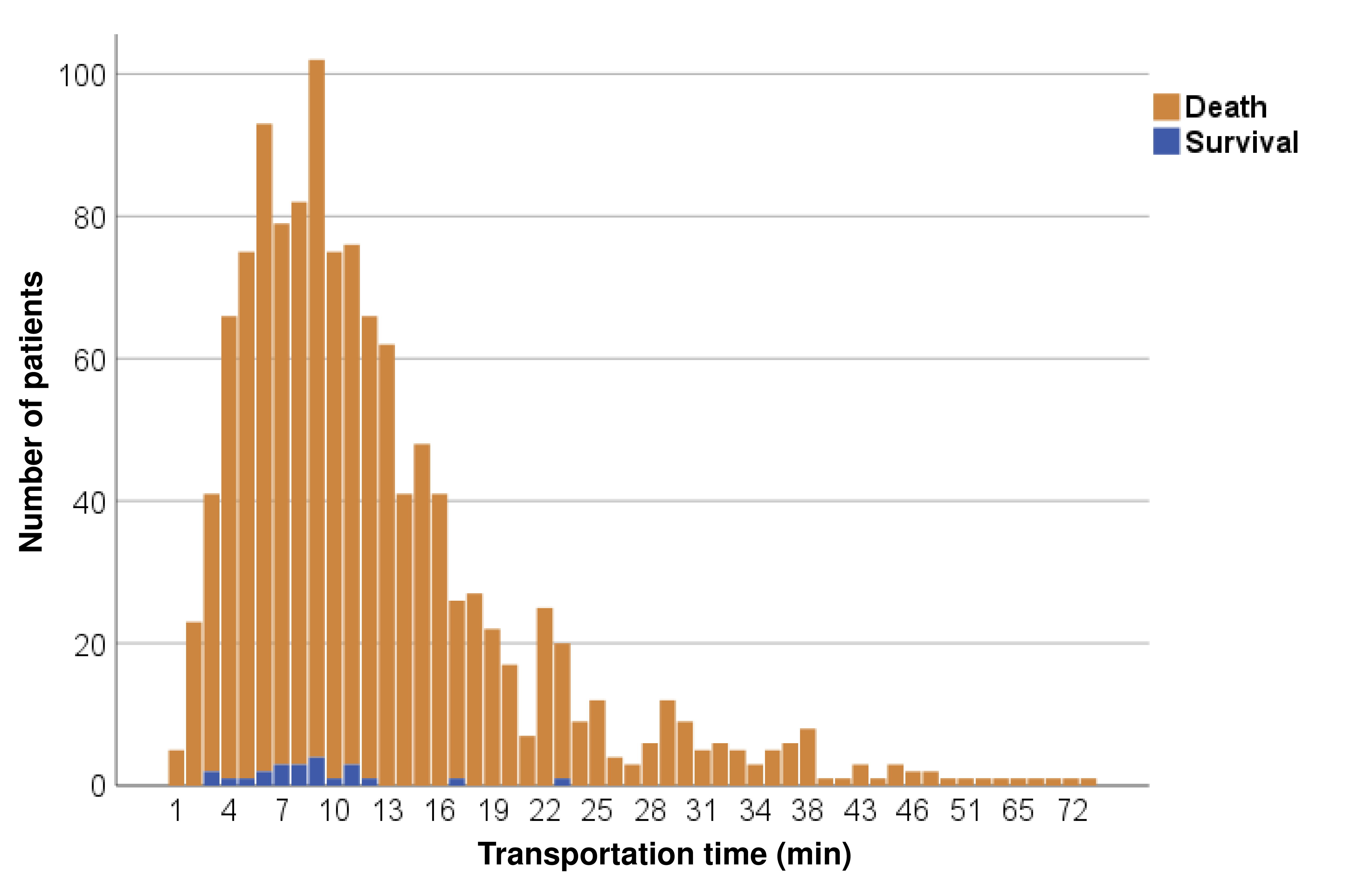

Supplement: Supplementary file 3 — Supplementary Figure S2. [file 41598_2023_43318_MOESM3_ESM.tiff]
