# Supplementary material for: Potential harms of emergency department thoracotomy in patients with persistent cardiac arrest following trauma: a nationwide observational study
Source: Sci Rep. 2023 Sep 25;13:16042. doi: 10.1038/s41598-023-43318-0 (PMC10520031; doi:10.1038/s41598-023-43318-0)
Supplement: Supplementary file 4 — Supplementary Table S1. [file 41598_2023_43318_MOESM4_ESM.docx]

| Table S1. Missing values | | | |
| --- | --- | --- | --- |
|  |  | Missing value | |
|  |  | N | % |
| Age |  | 0 | 0.0 |
| Sex |  | 6 | 0.5 |
| Mechanism of injury | | 0 | 0.0 |
| Comorbidity, Charlson index | | 0 | 0.0 |
| Activity of daily living, independent | | 101 | 7.8 |
| Prehospital procedure | |  |  |
|  | Intubation | 0 | 0.0 |
|  | Fluid administration | 0 | 0.0 |
|  | Transfusion | 0 | 0.0 |
| Physician presence at prehospital | | 0 | 0.0 |
| Signs of life | |  |  |
|  | At the scene | 0 | 0.0 |
|  | On hospital arrival | 118 | 9.2 |
| Transportation time | | 55 | 4.3 |
| Abbreviated Injury Scale | |  |  |
|  | Head/neck | 38 | 2.9 |
|  | Face | 38 | 2.9 |
|  | Chest | 38 | 2.9 |
|  | Abdomen | 38 | 2.9 |
|  | Extremity/pelvis | 38 | 2.9 |
|  | Body surface | 38 | 2.9 |
| Little's Missing Completely At Random test: p-value < 0.001 | | | |
