# Supplementary material for: Potential harms of emergency department thoracotomy in patients with persistent cardiac arrest following trauma: a nationwide observational study
Source: Sci Rep. 2023 Sep 25;13:16042. doi: 10.1038/s41598-023-43318-0 (PMC10520031; doi:10.1038/s41598-023-43318-0)
Supplement: Supplementary file 5 — Supplementary Table S2. [file 41598_2023_43318_MOESM5_ESM.docx]

| Table S2. Survival to discharge in sensitivity analyses | | | | | |  |
| --- | --- | --- | --- | --- | --- | --- |
|  |  | EDT | No EDT | OR | 95% CI |  |
| In-hospital mortality, *% (95% CI)* | |  |  |  |  |  |
|  | Generalized estimating equations |  |  | 0.08 | 0.01–0.71 |  |
|  | Multivariate logistic regression* |  |  | 0.06 | 0.01–0.76 |  |
|  | IPW with restriction** | 1.0% | 2.2% | 0.44 | 0.22–0.87 |  |
|  | IPW with original data (before missing value imputation) | 0.4% | 2.8% | 0.13 | 0.05–0.38 |  |
|  | IPW without injury severity*** | 1.0% | 2.5% | 0.41 | 0.21–0.78 |  |
| EDT, emergency department thoracotomy; OR, odds ratio; CI, confidence interval; and IPW, inverse probability weighting. *Frequency of EDT in each institution was entered into the model along with the same covariates for propensity score calculation. **Patients with 0.05–0.95 of propensity score were included. ***IPW with propensity score was repeated after excluding the injury severity from propensity score calculation. | | | | | |  |
|  |  |  |  |  |  |  |
|  |  |  |  |  |  |  |
|  |  |  |  |  |  |  |
|  |  |  |  |  |  |  |
